# Adverse event following immunization or vaccination in children in Minas Gerais: 2015 to 2020

**DOI:** 10.1590/1980-549720230056

**Published:** 2023-12-11

**Authors:** Sabrina Marteleto de Camargos, Maria Luisa Sena de Oliveira, Bianca Maria Oliveira Luvisaro, Thales Philipe Rodrigues da Silva, Janaina Fonseca Almeida Souza, Aline Mendes Vimieiro, Tércia Moreira Ribeiro da Silva, Fernanda Penido Matozinhos

**Affiliations:** I Universidade Federal de Minas Gerais, Escola de Enfermagem – Belo Horizonte (MG), Brazil.; II Universidade Federal de São Paulo, Escola Paulista de Enfermagem – São Paulo (SP), Brazil.; III Universidade Federal de Minas Gerais, School of Nursing, Postgraduate Program in Nursing – Belo Horizonte (MG), Brazil.; IV State Health Secretariat of Minas Gerais – Belo Horizonte (MG), Brazil.

**Keywords:** Immunization, Medication errors, Child, Patient safety, Public health surveillance, Communicable diseases, Imunização, Erros de medicação, Criança, Segurança do paciente, Vigilância em saúde pública, Doenças transmissíveis

## Abstract

**Objective:**

To describe adverse event following immunization or vaccination in children in Minas Gerais: 2015 to 2020, resulting from immunization errors in children from zero to nine years old.

**Methods:**

An ecological, descriptive study with a quantitative approach, based on event notifications available in the National Immunization Program Information System.

**Results:**

Among the 39,903,277 doses of immunobiologicals in children aged zero to nine, administered in the state of MG, 3,259 events of types of immunization errors were recorded, around 0.008% of the total and, of these, 91.86% did not result in adverse events and 56.02% were children under one year of age. The most frequent diagnosis was application outside the recommended age (29.12%). Among the manifestations, 71.91% were local and systemic, with fever being the most common (40.85%).

**Conclusion:**

The study demonstrated that immunization errors were rare and that most of them were not associated with adverse events, which reinforces the safety of the immunization process. This undoubtedly raises reflection on the need and relevance of continuing education for health professionals.

## RESUMO

**Objetivo:**

Descrever os eventos supostamente atribuíveis à imunização ou vacinação em crianças de Minas Gerais, de 2015 a 2020, decorrentes dos erros de imunização em crianças de 0 a 9 anos.

**Métodos:**

Estudo ecológico, descritivo, de abordagem quantitativa, fundamentado nas notificações de eventos disponíveis no Sistema de Informações do Programa Nacional de Imunizações.

**Resultados:**

Dentre os 39.903.277 de doses de imunobiológicos administrados em crianças de 0 a 9 anos, no estado de Minas Gerais, registraram-se 3.259 eventos do tipo erros de imunizaçõo, cerca de 0,008% do total e, destes, 91,86% não acarretaram eventos adversos e 56,02% ocorreram em crianças menores de 1 ano. O diagnóstico mais frequente foi a aplicação fora da idade recomendada (29,12%). Dentre as manifestações, 71,91% foram locais e, em relação às sistêmicas, a mais comum foi a febre (40,85%).

**Conclusão:**

O estudo demonstrou que os erros de imunização foram raros e a maioria deles não se associou aos eventos adversos, o que reitera a segurança no processo de imunização. Isso suscita, indubitavelmente, a reflexão sobre a necessidade e a relevância de educação permanente dos profissionais da saúde.

## INTRODUCTION

Childhood vaccination is one of the most important public health measures to avoid diseases preventable by immunization and reduce morbidity and mortality, especially among this population, which may be affected by many diseases, such as polio, measles, mumps, and rubella^1–3^. In this sense, the drop in vaccination coverage can lead to relevant public health issues, such as outbreaks and epidemics of diseases that have already been controlled or even eradicated^1–3^.

In the Brazilian scenario, a major epidemiological milestone was the sharp drop in infant mortality, especially due to the creation of the National Immunization Program (*Programa Nacional de Imunizações* – PNI) in 1973^4^. PNI's goal is to fully vaccinate Brazilians through an organized system, according to age range, specific calendars, and periodic campaigns^4^.

Currently, the Brazilian Unified Health System (*Sistema Único de Saúde* – SUS), through PNI, offers free of charge vaccines with epidemiological relevance for public health recommended by the World Health Organization (WHO)^1–4^. Brazil is considered a global reference in immunization, mainly due to its high vaccination coverage for several immunobiologicals, especially among children and young people^1–4^. However, there has been a decrease in vaccination coverage in recent years, which was heightened during the new coronavirus (COVID-19) pandemic, following the global trend^3^.

PNI prioritizes safe vaccination through the health surveillance system, which operates in all immunization processes, including pharmacovigilance5. This is essential to quickly detect and respond to events supposedly attributable to vaccination and/or immunization (ESAVI), reducing the risks to people's health and the negative impact on the immunization program^5^.

According to WHO, an ESAVI is any unwanted medical occurrence temporally associated with vaccination, without necessarily having a causal relationship with the use of a vaccine or other immunobiologicals^6^. ESAVIs can be classified according to the type, severity, and causality of manifestations^5,6^.

Regarding causality, these events are divided into five categories:

- product-related reaction;
- reaction related to the quality of vaccines;
- anxiety reaction related to immunization or response triggered by stress resulting from vaccination;
- coincidental or of unknown cause; and
- immunization errors.

In many countries, the majority of ESAVIs are related to immunization errors, therefore, when investigating causality, it is essential to assess the possibility of this error occurring^5,7^. When caused by an immunization error, ESAVI can be considered an isolated event or a set of events associated with vaccination^5,7^.

WHO defines immunization errors as any preventable event that can cause inappropriate use of immunobiologicals and/or harm to a patient^5^. It may be associated with professional practice, health products, procedures and systems, including prescription, verbal guidance, labeling, packaging and nomenclature of industrialized and manipulated products, dispensing, distribution, administration, education, monitoring, and use^5^. Immunization errors are, therefore, preventable through safe vaccination practices^5^.

In addition to possible harm to patients, such errors can negatively impact the population's confidence in vaccination, since confidence is related to ESAVI experiences and the credibility of institutions and health professionals^5,8^. Consequently, these events have the potential to lead to vaccine hesitancy and reduce vaccination coverage, harming the control of diseases preventable by immunization^5^.

Therefore, the investigation and identification of immunization errors are of interest to public health and can assist in changes regarding surveillance, service management, nursing performance in immunization services and professional qualification, improving the quality of care, prevention and reduction of the risk of harm to users^8^.

The present study aimed to describe the events supposedly attributable to immunization or vaccination in children aged 0 to 9 years, in Minas Gerais, in the years 2015 to 2020, resulting from immunization errors.

## METHODS

### Type of study

This is an ecological, descriptive study with a quantitative approach, based on ESAVI notifications registered in the PNI Information System (*Sistema de Informações do PNI* – SI-PNI).

### Location

Minas Gerais is made up of 853 municipalities spread over 586,513.993 km², with an estimated population of 21,411,923 inhabitants (2021)^9^. To better organize health care, the state was divided into 14 macro-regions, considering their demographic, socioeconomic, geographic, sanitary, and epidemiological particularities^10^: Central South, Central, Jequitinhonha, West, East, South, Southeast, North, Northwest, East South, Northeast, Vale do Aço, Triângulo do Sul, and Triângulo do Norte^10^.

### Selection criteria

As inclusion criteria, all notifications of immunization errors in children aged 0 to 9 years were considered, from January 1^st^, 2015 to December 31^st^, 2020 in Minas Gerais, Brazil.

The immunobiologicals studied were those recommended for this audience, which are offered by SUS and/or the private network: BCG; hepatitis B; inactivated polio — IPV, attenuated oral polio — OPV; human rotavirus — RV1; rotavirus pentavalent — RV5; diphtheria, tetanus, pertussis, hepatitis B, and *Haemophilus influenzae* B — DTP/HEPB/HIB (pentavalent); pneumococcal 10V; pneumococcal 13V; meningococcal C; meningococcal ACWY; yellow fever, measles and rubella (double viral); measles, mumps, rubella (triple viral); measles, mumps, rubella, and varicella (tetra viral); hepatitis, diphtheria, tetanus, and pertussis — DTP (triple bacterial); diphtheria, tetanus, and (acellular) pertussis for infants — DTaP (acellular triple bacterial); adult tetanus and diphtheria — Td (adult double); *Haemophilus influenzae* B — HIB; pneumococcal 23V; varicella (attenuated); trivalent influenza; diphtheria, tetanus, and (acellular) pertussis for infants, inactivated polio, *Haemophilus influenzae* B — DTaP/HIB/inactivated polio (quintuple acellular); diphtheria, tetanus, and (acellular) pertussis for infants, inactivated polio, hepatitis B, *Haemophilus influenzae* B — DTaP-IPV-HB/HIB (hexavalent acellular); diphtheria, tetanus, and adult (acellular) pertussis — DTaP (adult acellular triple bacterial)^11–13^.

For the exclusion criteria, notifications of ESAVIs not resulting from immunization errors and notifications of immunization errors related to the human papillomavirus (HPV) vaccine were considered, which begins for females at age 9, but extends to beyond the age range studied.

### Data organization

Data for this study were collected in January 2022, using ESAVI notifications resulting from immunization errors registered in SI-PNI; the variables selected for analysis were: immunization error, gender, age, type of immunobiological administered, health macro-region, route of administration, type of event, clinical manifestations, diagnosis, medical care, and case evolution.

### Data analysis and processing

A database was built with the help of Excel 2014 (https://products.office.com/) and the statistical package Statistical Software for professional (Stata), version 16.0, was used to analyze the data. Estimates of ESAVIs resulting from immunization errors were presented in proportions (%), according to year of occurrence, health macro-region of Minas Gerais, and immunobiological administered. The data were stratified according to immunization errors with and without adverse events.

To calculate the incidence rate of immunization errors per 100,000 doses administered, the numerator was considered to be the number of immunization errors with and without adverse events and, as the denominator, the number of doses administered to children in the age group per period and health macro-region^14^. The number of doses of each immunobiological was obtained from the Ministry of Health (MoH) website: pni.datasus.gov.br.

### Ethical aspects

The research was approved by the Ethics Committee of Universidade Federal de Minas Gerais in 2020, under opinion 4.134.126, complying with the requirements of Resolution CNS 466/2012^15^. As these are non-nominal public data, the signing of the Informed Consent was not required.

## RESULTS

From 2015 to 2020, 39,903,277 doses of immunobiologicals were administered to children aged 0 to 9 years in the state of Minas Gerais, Brazil. 3,259 events classified as immunization errors were recorded, which was equivalent to 0.008% of the total doses administered. As for gender, 50.32% were female children and with regard to age, 56.02% occurred in children under 1 year of age. Among the errors, 91.86% did not result in adverse events, however, 8.14% resulted in some type of adverse event.

Chart 1 shows that 2019 had the highest proportions of immunization errors with (25.28%) and without (33.93%) adverse events.

**Chart 1:** Proportion and incidence rate of immunization errors, according to absence and presence of events supposedly attributable to vaccination or immunization in children aged 0 to 9 years per 100,000 doses applied. Minas Gerais, Brazil, 2015–2020^4^.

Year	Total doses administered to children	Immunization errors without adverse events			Immunization errors with adverse events		
		n	%	IR	n	%	IR
2015	6,976,441	154	5.14	2.20	42	15.85	0.60
2016	6,730,976	260	8.68	3.86	42	15.85	0.62
2017	6,307,137	325	10.86	5.15	39	14.72	0.61
2018	6,969,232	481	16.07	6.90	32	12.08	0.45
2019	6,279,050	1,016	33.93	16.18	67	25.28	1.06
2020	6,640,441	758	25.32	11.41	43	16.23	0.64
Total	39,903,277	2,994	100	7.50	265	100	0.66

IR: incidence rate of immunization errors.

Regarding the incidence rate, it was found that immunization errors without adverse events, in the period studied, resulted in 7.50/100,000 doses applied, and with adverse events, in 0.66/100,000. In 2019, there was the highest incidence rate of ESAVIs resulting from immunization errors without (16.18/100,000 doses applied) and with adverse events (1.06/100,000 doses applied) (Chart 1).

Regarding the health macro-regions of Minas Gerais, Triângulo do Norte had the highest proportion of immunization errors without adverse events (25.12%). The Central region had the highest percentage of immunization errors with adverse events (44.91%). With reference to the incidence rate, the highest one recorded without ESAVI (27.27/100,000 doses administered) was in Triângulo do Norte and with ESAVI (1.3/100,000 doses administered), in Leste do Sul (Chart 2).

**Chart 2:** Distribution of events supposedly attributable to vaccination or immunization in children aged 0 to 9 years in the macro-regions of Minas Gerais, 2015–2020^4^.

Macro-region	Doses administered	Without adverse event		With adverse event	
		n (%)	IR	n (%)	IR
Central	12,483,015	548 (18.30)	4.38	119 (44.91)	0.95
Central South	1,441,987	148 (4.94)	10.26	6 (2.26)	0.41
Jequitinhonha	759,491	64 (2.14)	8.42	4 (1.51)	0.52
East	1,373,887	26 (0.87)	1.89	1 (0.38)	0.07
Leste do sul	1,297,856	73 (2.44)	5.62	17 (6.42)	1.30
Northeast	1,594,213	29 (0.97)	1.81	3 (1.13)	0.18
Northwest	1,356,166	25 (0.84)	1.84	8 (3.02)	0.58
North	3,315,261	243 (8.12)	7.32	22 (8.30)	0.66
West	2,312,138	262 (8.75)	11.33	14 (5.28)	0.60
Southeast	2,826,783	135 (4.51)	4.77	5 (1.89)	0.17
South	5,215,413	345 (11.52)	6.61	23 (8.68)	0.44
Triângulo do Norte	2,756,773	752 (25.12)	27.27	18 (6.79)	0.65
Triângulo do Sul	1,454,517	180 (6.01)	12.37	17 (6.42)	1.16
Vale do Aço	1,715,777	164 (5.48)	9.55	8 (3.02)	0.46
Total	39,903,277	2,994	7.50	265	0.66

IR: incidence rate of immunization errors.

In relation to the recommended immunobiologicals, the triple viral (measles, mumps, and rubella) was the one with the highest percentage of immunization errors without ESAVI (17.27%), followed by the polio (attenuated) — OPV (8.62 %) and human rotavirus (8.60%) vaccines. Regarding immunization errors with ESAVI, BCG had the highest percentage (15.32%), followed by DTP/HEPB/HIB (diphtheria, tetanus, pertussis, hepatitis B, and *Haemophilus influenzae* B) (11.59%) and by DTP (diphtheria, tetanus, and pertussis) (11.38%) (Chart 3).

**Chart 3:** Immunobiological immunization error according to the absence and presence of events supposedly attributable to vaccination or immunization in children, in accordance with recommendations from the Brazilian Ministry of Health. Minas Gerais, Brazil, 2015–2020.

Immunobiological	Without adverse event		With adverse event	
	n	%	n	%
BCG	252	4.84	74	15.32
Hepatitis B	214	4.11	14	2.89
Diphtheria, tetanus, and (acellular) pertussis for infants — DTaP	13	0.25	2	0.41
Diphtheria, tetanus, and pertussis — DTP (triple bacterial)	305	5.86	55	11.38
*Haemophilus influenzae* B — HIB	15	0.28	2	0.41
(Inactivated) polio — IPV	357	6.86	33	6.83
Human rotavirus (attenuated)	447	8.60	42	8.69
Rotavirus pentavalent	2	0.03	-	-
Pneumococcal 10V	373	7.17	31	6.41
Pneumococcal 13V	1	0.01	-	-
Diphtheria, tetanus, and (acellular) pertussis for infants, inactivated polio, *Haemophilus influenzae* B — DTaP/HIB/inactivepolio (quintuple)	2	0.03	-	-
Diphtheria, tetanus, pertussis, hepatitis B, and *Haemophilus influenzae* B — DTP/HEPB/HIB (pentavalent)	398	7.65	56	11.59
Diphtheria, tetanus, and (acellular) pertussis for infants, inactivated polio, hepatitis B, *Haemophilus influenzae* B — DTaP-IPV-HB/HIB (hexavalent)	4	0.07	-	-
Meningococcal C	255	4.90	19	3.93
Meningococcal ACWY	10	0.19	-	-
Trivalent influenza	212	4.07	37	7.66
(attenuated) polio OPV	448	8.62	28	5.79
Yellow fever	311	5.98	18	3.72
Hepatitis A	160	3.07	16	3.31
Measles, mumps, rubella – MMR (triple viral)	898	17.27	34	7.03
Measles, mumps, rubella, and varicella — MMRV (tetra viral)	24	0.46	1	0.20
Varicella	311	5.98	14	2.89
Pneumococcal 23V	16	0.30	-	-
Diphtheria, tetanus, and adult (acellular) pertussis — DTaP	127	2.44	6	1.24
Diphtheria and tetanus — DT (adult double)	39	0.75	-	-
Measles and rubella — MR (double viral)	3	0.05	1	0.20
Total	5,197	100	483	100

The most frequent immunization errors without adverse events were the application outside the recommended age (29.12%), the change of immunobiologicals (13.62%), and the application of an immunobiological (laboratory) not recommended for the age (10.64 %). With adverse events, the most frequent error was programmatic (9.05%). Application past expiration date, diluent/dilution error, wrong dose, and application of an immunobiological (laboratory) not recommended for the age were not associated with any immunization errors with adverse events. Furthermore, 24.38% of immunization errors without adverse events and 87.54% with adverse events were considered to be without information (Chart 4).

**Chart 4:** Diagnosis according to the absence and presence of events supposedly attributable to vaccination or immunization in children. Minas Gerais, Brazil, 2015–2020^4^.

Diagnostics	Immunization error without adverse event		Immunization error with adverse event	
	n	%	n	%
Programmatic error	72	2.40	24	9.05
Swapped immunobiologicals	408	13.62	2	0.75
Administration beyond the recommended age	872	29.12	6	2.26
Administration of expired products	294	9.81	0	0
Wrong route of administration	36	1.20	1	0.37
Diluent/dilution error	85	2.83	0	0
Wrong dose	178	5.94	0	0
Administration of immunobiological (laboratory) not recommended for age	319	10.65	0	0
No information	730	24.38	232	87.54
Total	2,994	100	265	100

Most manifestations resulting from immunization errors were local (71.91%), among which the majority (63.06%) were pain (19.76%), heat (15.47%), edema (14.76%), and erythema or flushing (13.09%). Regarding systemic manifestations, the most common was fever (40.85%).

It is noteworthy that 99.46% of notifications of immunization errors without adverse events did not meet the medical care variable, 0.93% did not receive care and 0.1%, did. Regarding immunization errors with adverse events, 53.58% received medical care, 27.16% did not need care, and in 19.24% of notifications, this variable was ignored.

Considering the evolution of the case, there was no recorded information in 99.69% of immunization errors without an adverse event and in 49.07% of errors with an adverse event. Immunization errors with adverse events were cured without sequelae in 48.63% of cases and with sequelae in 2.27% of them.

## DISCUSSION

During the period studied, it was observed that immunization errors were rare events, considering the total number of doses applied, which reinforces the safety of the vaccination process. However, the incidence rate of ESAVIs resulting from immunization errors, from 2015 to 2019, increased. This can be justified by the increase in notifications resulting from the expansion of the surveillance and notification system, the increase in failures in nursing care or updates to the vaccination calendar, which occurred in the face of new scientific evidence and the improvement of technologies without adequate team training^8^. Therefore, the multidisciplinary team needs ongoing education to update protocols and knowledge, improving the quality of care^8^.

In 2020, a significant drop in the incidence rate of ESAVIs resulting from immunization errors was identified compared to 2019, which may be related to the sharp drop in vaccination coverage during the COVID-19 pandemic^16^. The mean vaccination coverage for BCG, hepatitis B, human rotavirus, meningococcal C, pentavalent, pneumococcal 10V, polio, pneumococcal 10V (first booster), meningococcal C (first booster), and triple viral (first dose) fell by approximately 11.10% from 2019 to 2020, reaching its lowest value in the period from 2013 to 2020^16^. Therefore, in 2020, none of these vaccines reached the proposed coverage goal^16^.

In relation to age, children under 1 year of age were the most affected by immunization errors (56.02%), which may be favored by uneasiness at the time of vaccination, poor skills of some professionals, greater frequency of this population in health units, which provides opportunities for notification, and due to the highest concentration of immunobiologicals administered in this age group, totaling nine immunobiologicals according to the Ministry of Health's vaccination calendar^8,17^.

This large quantity of immunobiologicals can lead to a series of errors, such as timing, swapping immunobiologicals, wrong route of administration, and wrong dose^18^. Therefore, one must always check whether patients, vaccine, dose, age range, preparation, route of administration and registration are correct^19^.

Regarding macro-regions, there is an uneven distribution of immunization error incidence rates, presenting data that suggest underreporting of these events. This may have been influenced by the implementation of the SI-ESAVI notification system, which did not occur simultaneously in all health macro-regions, as well as access to computers in immunization rooms^20^. Furthermore, there may have been a lack of knowledge about the need to notify cases, in addition to the difficulty of registering them in a short period of time and the slowness of the notification system on computers^21^.

By analyzing the types of immunobiologicals, the highest proportions of immunization errors with ESAVI were related to the BCG vaccine, followed by DTP/HEPB/HIB (diphtheria, tetanus, pertussis, hepatitis B, and *Haemophilus influenzae* B) and DTP (diphtheria, tetanus, and pertussis), as shown in other studies on immunization errors^8,22^.

The BCG vaccine contains a suspension of live attenuated bacteria and is applied intradermally^23^. This route of administration requires specific technique and training from the vaccinator, as a deep application can lead to adverse events, such as abscesses, ulcers larger than 1 cm and lymphadenopathies^8^. The DTP/HEPB/HIB and DTP vaccines contain aluminum hydroxide as adjuvant in their composition, which possibly favors local inflammation reactions and adverse events^7,8^. However, it is important to highlight that most of these events are mild and spontaneously resolving, reiterating that the benefits of immunization are substantially greater than the risks^7,8^.

In relation to the cold chain, changes in temperature can impair the effectiveness of the vaccine and lead to manifestations arising from changes in the physical state of the immunobiologicals, such as the aggregation of aluminum-based excipients^5^. Furthermore, some studies indicate that the improper application, via subcutaneous route, of vaccines containing the aluminum hydroxide adjuvant, may be associated with cold abscesses^5,7,8^. Hence the importance of applying the correct administration technique in the indicated route and of homogenizing the vaccine vial before aspirating, thus avoiding the accumulation of this substance at the bottom of the vial, as well as ensuring ideal storage conditions^5,7,8^.

The most frequent errors without adverse events were: application outside the recommended age range and swapped immunobiologicals. These may be related to non-verification of information, lack of professional knowledge, organization of the vaccination room, vaccine labels, and changes to the vaccination schedule without adequate training of professionals^17,18^. Errors can be avoided through ongoing education about the vaccines included in the calendar, presence of material to consult the standards, use of only one brand of the same immunobiological and storage separately from immunobiologicals with similar names or bottles^17,18^. Furthermore, the habit of discussing errors that have occurred with healthcare professionals should be adopted in order to highlight points for improvement and to propose solutions^17,18^.

Regarding immunization errors with adverse events, most notifications did not present information and, when they did, the type of error was not correctly specified. One of the factors that influence many professionals to fail to report immunization errors or ESAVIs is the fear of being reprimanded^8,17^. Therefore, health institutions must guarantee professionals are safe to do so and awareness that they are contributing to avoiding errors^8,17^.

The need to improve continuing education demonstrated in this study corroborates other research, which identified limitations or absence of professional nurses’ performance in the vaccination room. In this way, many failures in the immunization process could be avoided through the direct action of professionals, including permanent education, on topics such as good vaccination practices, inclusion of the patient in the immunization process and monitoring of adverse reactions, supervision of activities, managing possible adverse reactions and monitoring the conservation of immunobiologicals^24,25^.

As for the manifestations resulting from the error, the majority were not serious, being characterized as pain, edema, flushing or fever. It is worth highlighting that, when these manifestations are not caused by immunization errors, they are inflammatory reactions possibly related to the physiochemical properties of immunobiologicals, such as adjuvants and preservatives^22,24–26^.

With regard to medical care and the evolution of immunization errors without ESAVI, the value found demonstrates that the notification forms are not being filled out properly, which significantly interferes with the quality of the information generated by SI-ESAVI^21^.

Regarding errors with adverse events, the majority progressed to cure without sequelae, which refers to the low severity of these episodes^21^. However, the lack of seriousness should not inhibit the notification and investigation of the case with medical monitoring and laboratory tests^21^.

This study used the SI-SIPNI/SI-ESAVI database, therefore, it presented limitations related to the use of secondary data, such as underreporting of ESAVIs resulting from immunization errors and inadequate completion of records. However, considering the amount of data collected, the work made important contributions to the investigation of the causes of ESAVIs resulting from errors, which makes it possible to develop behaviors that contribute to safe nursing practice in the vaccination room and, consequently, the maintenance of credibility of PNI for the population.

This study demonstrated that immunization errors were rare (around 0.008%) and that the majority were not associated with adverse events, reiterating the safety of the immunization process. Furthermore, it raised reflection on the need to permanently educate health professionals, promote a culture of patient safety and increase awareness that investigating immunization errors can clarify their causes and develop strategies to avoid them, taking into account they are preventable through safe practices, they are therefore opportunities to improve work processes.
